# Measurements of complex refractive index change of photoactive yellow protein over a wide wavelength range using hyperspectral quantitative phase imaging

**DOI:** 10.1038/s41598-018-21403-z

**Published:** 2018-02-15

**Authors:** KyeoReh Lee, Youngmin Kim, JaeHwang Jung, Hyotcherl Ihee, YongKeun Park

**Affiliations:** 1 Department of Physics, Korea Advanced Institute of Science and Technology, Daejeon, 34141 Republic of Korea; 2 Center for Nanomaterials and Chemical Reactions, Institute for Basic Science (IBS), Daejeon, 34141 Republic of Korea; 3 Department of Chemistry, Korea Advanced Institute of Science and Technology, Daejeon, 34141 Republic of Korea

## Abstract

A novel optical holographic technique is presented to simultaneously measure both the real and imaginary components of the complex refractive index (CRI) of a protein solution over a wide visible wavelength range. Quantitative phase imaging was employed to precisely measure the optical field transmitted from a protein solution, from which the CRIs of the protein solution were retrieved using the Fourier light scattering technique. Using this method, we characterized the CRIs of the two dominant structural states of a photoactive yellow protein solution over a broad wavelength range (461–582 nm). The significant CRI deviation between the two structural states was quantified and analysed. The results of both states show the similar overall shape of the expected *r*RI obtained from the Kramers–Kronig relations.

## Introduction

Light-matter interaction is key to the characterization of a sample and is performed using a diverse range of existing methodologies, including visual inspection, scattering analysis, microscopy, and spectroscopy. The light-matter interaction represents the composite of the electric and magnetic responses of material. It can be quantified by a single parameter called the complex refractive index (CRI) described by the expression

1 $$\,{\rm{CRI}}(\lambda )=\sqrt{[1+{\chi }_{e}(\lambda )][1+{\chi }_{m}(\lambda )]}=n(\lambda )+i\kappa (\lambda ).$$

Here, *n* and *κ* are the real and imaginary RIs, which describe reflection and transmission by bulk media, *χ*_*e*_ and *χ*_*m*_ are the electric and magnetic susceptibilities, and *λ* is the wavelength of light, respectively. The real and the imaginary parts (*n* and *κ*) have extensively used separately as refractive index and extinction coefficient, respectively. As in Eq. (1), the CRI is a complex value that characterizes the electromagnetic response of the matter. The CRI has been importantly utilized in physical chemistry, analytical chemistry, and structural biology, where light is used as a probe^1–7^.

In particular, direct measurement of the CRIs of proteins is of great interest to biophysical chemists and because the CRI of a protein is related to its chromophore structure and chromophore environment. As an example, circular dichroism spectroscopy, which analyses the CRI difference between the two circular polarizations of light, has been utilized to determine the secondary structures of proteins^8^ and ultraviolet-visible spectrophotometry, which measures the imaginary part of the CRI (molar extinction coefficient) has been utilized to determine the protein concentration or property^9^.

Unfortunately, despite their powerful molecular characterization capabilities, direct measurement of the CRIs of protein solutions over a wide range of wavelengths has been hindered by limitations in existing instruments. Generally, the real part (*r*RI) and imaginary part (*i*RI) of the CRI can be separately measured using a reflectometer and an absorption spectrometer, respectively; however, the simultaneous measurement has been hardly realized. Simultaneous CRI measurement would be highly preferable for protein studies with the capability of performing spectroscopic measurements because the proteins are sensitive to surrounding environment such as temperature, pH, and ion concentrations, which may be perturbed during each individual measurement^10–13^. Simultaneous CRI measurement is crucial especially for proteins that undergo conformational changes upon specific modulations because CRI can be used as a non-invasive and quantitative reporter for the alterations in proteins. Alternatively, Kramers–Kronig (K–K) relations can be utilized to estimate the rRI from the measured *i*RI or vice versa. However, the K–K relations have a severe quantification problem unless the detection wavelength range is sufficiently broad. Spectroscopic ellipsometry can also be used to measure the CRI components simultaneously; however, the target sample needs to be a uniform thin film of known thickness^14^. Thus, generally, the ellipsometry technique is primarily used to observe protein adsorption kinetics, rather than for the optical characterization of protein solutions^15^.

In order to simultaneously measure the CRI components of a protein solution, we employed quantitative phase imaging (QPI) for measurement, and Fourier transform light scattering (FTLS) techniques^16^ for analysis of the QPI data. QPI uses the principle of holography to quantitatively and precisely measure both the optical attenuation and phase delay images when light passes through the sample. In other words, QPI uses the optical field image of a sample, which is directly related to the sample’s CRI^17,18^. The optical field images of both non-biological and biological samples, including colloidal particles^19,20^, red blood cells^21,22^, neurons^23,24^, and tissues^25,26^, have been successfully measured using QPI techniques. FTLS converts the measured optical field images into light scattering information using numerical far-field propagation^16^. Unlike conventional scattering analysis of bulk samples, FTLS specializes in light scattering analysis of individual microscopic samples, such as microspheres^27–29^, colloidal particles^19^, red blood cells^21,30,31^, bacteria^32,33^, and tissues^16^. In this work, we induce light scattering inside the protein solution by immersing a known microsphere in the target solution. Because the light scattering information is highly sensitive to the CRI of the surrounding medium, the CRI of the protein solution can be calculated precisely from the light scattering information using Mie theory^34^.

Using this method, we measured the CRI as a function of wavelengths of both a photoactive yellow protein (PYP) and its excited state by illumination with a blue light^35–38^. PYP is a protein related to phototaxis signal transduction in *Halorhodospira halophila*. PYP has been extensively investigated using various spectroscopic techniques because it serves as an important model system for the study of the relation between structural change and the signal transduction process^39–46^. PYP is also of interest as a photoresponsive module with potential as an optogenetic tool or artificial protein machine because of its small molecular size (125 amino acids, 14 kDa), high solubility, and photoactivity coupled with large structural change. The chromophore of PYP, deprotonated p-coumaric acid, absorbs blue light and demonstrates the conformational change cycle. When irradiated with blue light, the ground state of PYP (*pG*, *λ*_max_ = 446 nm; see Supplementary Fig. S3), which has trans-*p*-coumaric acid as a chromophore, is photo-isomerized to red-shifted intermediate *pR*_1_ and *pR*_2_ states (*λ*_max_ = 465 nm). The *pR*_1_ and *pR*_2_ decay with timescales on the order of microseconds and hundreds of microseconds, respectively, into blue-shifted intermediate *pB*_1_, which further transforms into another blue-shifted intermediate *pB*_2_ (*λ*_max_ = 355 nm), with cis-*p*-coumaric acid as the chromophore^47^. Finally, the *pB*_2_, which is the putative signaling state of PYP, returns to the *pG* state on hundreds of milliseconds time scale, and the photocycle ends. Amongst the several other intermediates of PYP, *pB*_2_ shows the largest conformational change and the longest relaxation time during the photocycle^42–44,46^. Because the relaxation times of the other intermediate states between *pG* and *pB*_2_ are comparatively short, the time-averaged excited PYP state can be simply regarded as a pure *pB*_2_ state. Therefore, for the rest of this paper, we simply denote the ground and excited states of PYP as the *pG* and *pB* states, respectively.

Though the absorption spectrum or *i*RI has been one of the most popular spectroscopic probes, the *r*RI of PYP has not been extensively studied and only estimated qualitatively^48^. In this work, we precisely quantify the *r*RI and *i*RI simultaneously for both the *pG* and *pB* states of PYP solution, using the proposed holographic techniques.

## Results

### Experimental procedure

**Figure 1:**
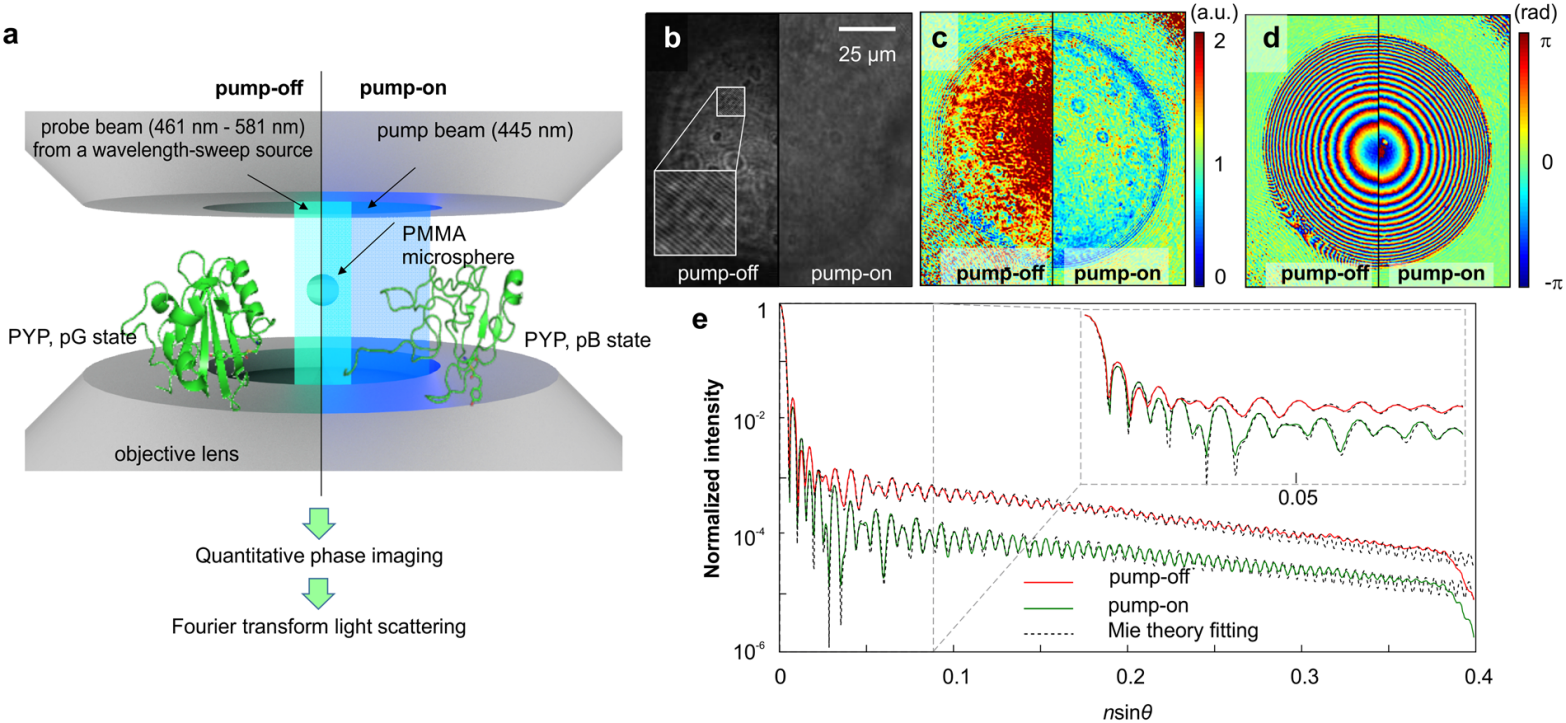
Experimental procedure for measuring the CRI of PYP solution. (**a**) A conceptual schematic of the measurements. The optical field of a microsphere immersed in PYP solution is obtained over a broad range of visible wavelengths; *left*, in the absence of a pump beam (pump-off); *right*, in the presence of a pump beam (pump-on), where most PYP converts to the excited state (*pB*). (**b**) Raw hologram of a PYP solution without (left) and with (right) the pump beam. (**c**) Measured amplitude and (**d**) phase images of microspheres immersed in the PYP solution. (**e**) Retrieved FTLS results of the PYP solution in pump-off (red) and pump-on (green) cases. The solid and dotted lines represent the experimental results and theoretical (Mie theory) fitting, respectively. *Inset*, FLTS results within the smaller scattering angle range.

In order to obtain the CRI of PYP, we first measured the multi-wavelength light field images of a 100-μm-diameter polymethyl methacrylate (PMMA) microsphere immersed in PYP solution (Fig. 1). The illumination wavelength for the optical field measurement (probe beam) is systemically scanned over a broad visible spectral range. The centre wavelength and bandwidth of the probe beams are defined by the prism, lens, and pinhole used in the illumination^12^. For optical field measurements, a quantitative phase imaging unit (QPIU)^49^ was implemented. The QPIU is a common-path full-field interferometer that uses the principle of lateral shearing interferometry (see Supplementary Fig. S1 online for the detailed optical setup).

The measurements were performed with and without the pump beam (445 nm peak; M455L3, Thorlabs Inc.) to measure the CRI of both the *pG* and *pB* states. To ensure state equilibrium of the PYP solution, we included sufficient idle time (>10 seconds) after switching the LED on or off before taking holograms. For each state, we obtained holograms at eleven different wavelengths ranging from 461 to 582 nm. The wavelengths were carefully selected to fully characterize the CRI of the PYP solution (see Supplementary Table S1 and Fig. S2 for detailed probe beam specifications). The nonlinear effects from the probe beam were negligible, and the intensities of probe beams were normalized in the data analysing process.

From each measured raw holographic image (Fig. 1b), the amplitude and phase images of the immersed microsphere were obtained with a conventional field retrieval algorithm (Fig. 1c,d)^50^. The retrieved light field images were converted into angle-resolved light scattering plots using FTLS, as shown in Fig. 1e. In FTLS, the measured optical field of a sample is numerically propagated to the far-field, which creates a direct 2D Fourier transformation of the optical field information of the sample. The angle-resolved light scattering plots are achieved by azimuthally averaging the 2D light scattering patterns, which greatly increase the signal-to-noise ratio (SNR) of the measurements. As the spatial analogous to Fourier-transform infrared spectroscopy, FTLS provides an unprecedented SNR in measuring scattered light signals owing to Fellgett’s advantage^51^. The azimuthal averaging is possible because the imaging target (a microsphere) is azimuthally symmetric. Then, the CRI was extracted by fitting the obtained FTLS results to the Mie scattering theory, which is the exact solution to Maxwell’s equations for light scattering from homogeneous spheres^52,53^.

### CRI of PYP solutions in *pG* and *pB* states

**Figure 2:**
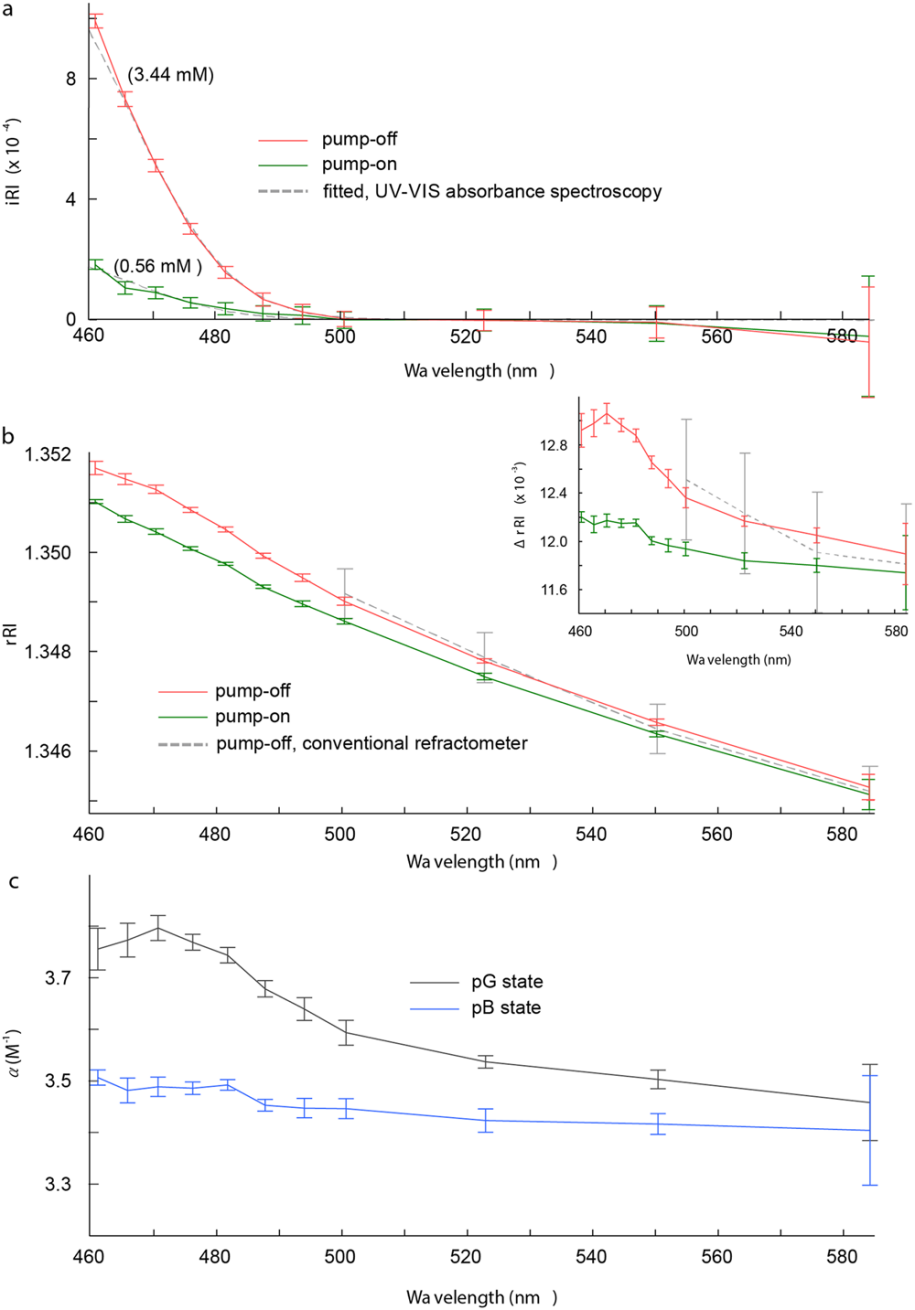
Measured CRI and refractive index increment of PYP solution. (**a**) The red line represents the *i*RI value of the PYP solution in the absence of pump beam illumination (pump-off). The green line represents the *i*RI value of the PYP solution in the presence of pump beam illumination (pump-on). The grey dotted line represents the fitted graphs of both of these values, based on Eq. (2). Corresponding concentrations of the ground state PYP are indicated in brackets on each line. (**b**) The *r*RI values of the PYP solution in the pump-off (red) and pump-on (green) environments. The grey dotted line represents the *r*RI result for a pump-off PYP solution obtained from a conventional refractometer for comparison. The inset shows the result after subtracting the *r*RI of distilled water from the PYP solution. (**c**) Refractive index increment (α) of *pG* (black) and *pB* (blue) states. The error bars indicate the standard deviation from five measurements with different microspheres immersed in identical PYP solutions.

The CRI values of both the *pG* and *pB* states of the PYP solution are shown in Fig. 2. The precision or standard deviation of the proposed method at each wavelength is depicted by the error bars. The mean precisions of the *r*RI and *i*RI are 8.2 × 10^−5^, and 4.2 × 10^−5^, respectively. The decrease in accuracy with increasing wavelength is caused by the bandwidth widening of the probe beam, which reduces the interference efficiency.

The *i*RI of the PYP decreases monotonically as the wavelength increases (Fig. 2a) and converges to zero for wavelengths longer than 500 nm. The *i*RI values in the presence of the pump beam (pump−on case) are approximately five times smaller than those of the PYP in the absence of the pump beam (pump−off case). The significant decrease in the *i*RI in the pump-on case indicates a PYP population transition from the *pG* to the *pB* state. Since the absorbance of *pB* is negligible for the current wavelength range (see Supplementary Fig. S3), we deduce that the non-zero *i*RI in the pump-on case is caused by the presence of a *pG* population. Therefore, the molecular density or concentration of *pG* (*ρ*_*pG*_) can be determined simply by

2 $$\,i{\rm{RI}}({\rho }_{pG},{\lambda }_{b})=\frac{{\rm{l}}{\rm{n}}(10)}{4\pi }{\rho }_{pG}{\lambda }_{b}\varepsilon ({\lambda }_{b}),$$

where *λ*_*b*_ is the wavelength of the probe beam and *ε* is the known molecular extinction coefficient of *pG*. The *ρ*_*pG*_ were measured as 3.44 ± 0.1 mM and 0.56 ± 0.1 mM for the pump−off and pump−on cases, respectively (Fig. 2a). Thus, the concentration of the *pB* state is found to be 2.88 ± 0.1 mM, and the *pB* population ratio (*R*_*pB*_ = amount of *pB*/amount of *pG*) is 0.837 ± 0.035. Because a continuous light source was used as the pumping source, *R*_*pB*_ = 1 is not achievable in this two-state equilibrium system^54^. A previous report that used LEDs for continuous illumination showed a similar *R*_*pB*_^55^.

The *r*RI values of the PYP decrease monotonically as the wavelength increases (Fig. 2b), which is a general phenomenon occurring in protein solutions due to the *r*RI of water. For a wavelength ranging from 461–582 nm, the *r*RI in the pump−off case decreased from 1.3518 to 1.3452, and the *r*RI in the pump−on case decreased from 1.3510 to 1.3450. The pump−on case shows lower *r*RI values than the pump−off case for the entire wavelength range. Unlike the *i*RI values, the quantitative *r*RI values have not been reported previously. Therefore, we verified the measured *r*RI values using a conventional refractometer (R-5000, ATAGO Co., Ltd). The verification was conducted with an identically prepared PYP solution in the pump-off case. The *r*RI values cannot be measured for short wavelength probes, owing to their lower intensities. The verification results are shown by the dotted grey lines in Fig. 2b, where the error bars indicate the minimum scale of the refractometer.

Similar to the *i*RI in Eq. (2), the *r*RI is also a function of molecular density. In order to characterize the PYP independent of the molecular density, we calculated the refractive index increment; that is, the density derivative of the real RI ($\partial r{\rm{RI}}/\partial \rho$). The refractive index increment of the PYP in the *pG* and *pB* states can be calculated individually using the linear equation:

3 $$\,[\begin{array}{c}r{{\rm{RI}}}_{pump-off}(\lambda )\\ r{{\rm{RI}}}_{pump-on}(\lambda )\end{array}]=(\begin{array}{cc}0 & {\rho }_{pG,pump-off}\\ {\rho }_{pB,pump-on} & {\rho }_{pG,pump-on}\end{array})[\begin{array}{c}{\alpha }_{pB}(\lambda )\\ {\alpha }_{pG}(\lambda )\end{array}]+r{{\rm{RI}}}_{{H}_{2}O}(\lambda ),$$

where *ρ*_*pG*_ and *ρ*_*pB*_ correspond to the molecular densities of the *pG* and *pB* states in the pump-on or pump–off cases, as denoted by the subscript; ${\alpha }_{pG}$ and ${\alpha }_{pB}$ are the refractive index increments ($\partial r{\rm{RI}}/\partial \rho$) of the PYP in the *pG* and *pB* states, respectively; $r{{\rm{RI}}}_{pump-off}$ and $r{{\rm{RI}}}_{pump-on}$ are the *r*RI values of the PYP solution in the pump-off and pump-on cases, respectively; and $r{{\rm{RI}}}_{{H}_{2}O}$ is the known *r*RI of distilled water^56^. The results are shown in Fig. 2c and tabulated in Supplementary Table S2. The difference between ${\alpha }_{pG}$ and ${\alpha }_{pB}$ was maximized (approx. 0.3 M^−1^) at 470 nm, and decreased as the wavelength increased.

The *pB* population ratio (*R*_*pB*_) of a state is related to the kinetics of the transition between the *pG* and *pB* states of the PYP. The relaxation time *τ* of the *pB* state to *pG* state can be obtained from the measured *R*_*pB*_ with the following equation (see Supplementary Information for details)^57,58^.

4 $$\frac{1}{\tau }=\,\mathrm{ln}(10)(\frac{1-{R}_{pB}}{{R}_{pB}})\frac{\varphi }{{N}_{A}hc}\int \varepsilon ({\lambda }_{P}){\lambda }_{P}\frac{\partial {I}_{P}}{\partial {\lambda }_{P}}d{\lambda }_{P},$$

where *h* is Planck’s constant, *N*_*A*_ is Avogadro’s constant, *λ*_*P*_ is the wavelength of the pump light, *ϕ* is the photocycle quantum yield of PYP, and ∂*I*_*P*_*/*∂*λ*_*P*_ is the spectral density of the pump beam. Inserting *ϕ* = 0.35 from the literature^59,60^ and ∂*I*_*P*_/∂*λ*_*P*_ (see Supplementary Fig. S2) and *R*_*pB*_ = 0.837 ± 0.035 from the measurement, *τ* was calculated to be 77 ± 27 ms. We note that the relaxation time determined here is smaller than those reported by typical time-resolved pump-probe experiments (0.15–2 s)^44,61,62^. The discrepancy may be related to the different modes of data collection (continuous illumination vs. pump-probe), but the exact origin is not clear at this stage. The accuracy of this calculated *τ* is mainly determined from the uncertainty of *R*_*pB*_ due to the high *R*_*pB*_ sensitivity of *τ* in Eq. (4).

## Discussion

The deviation in the *i*RI between the two structural states of the PYP is to be expected caused by the well-known different extinction coefficients between two states. The *r*RI, however, is a quantity that relates to the mass or density of the material^63,64^. Because only conformational change, not mass variation, occurred when the pump beam was turned on or off, the deviation in the *r*RI can be considered to arise from density variations resulting from the conformational change.

**Figure 3:**
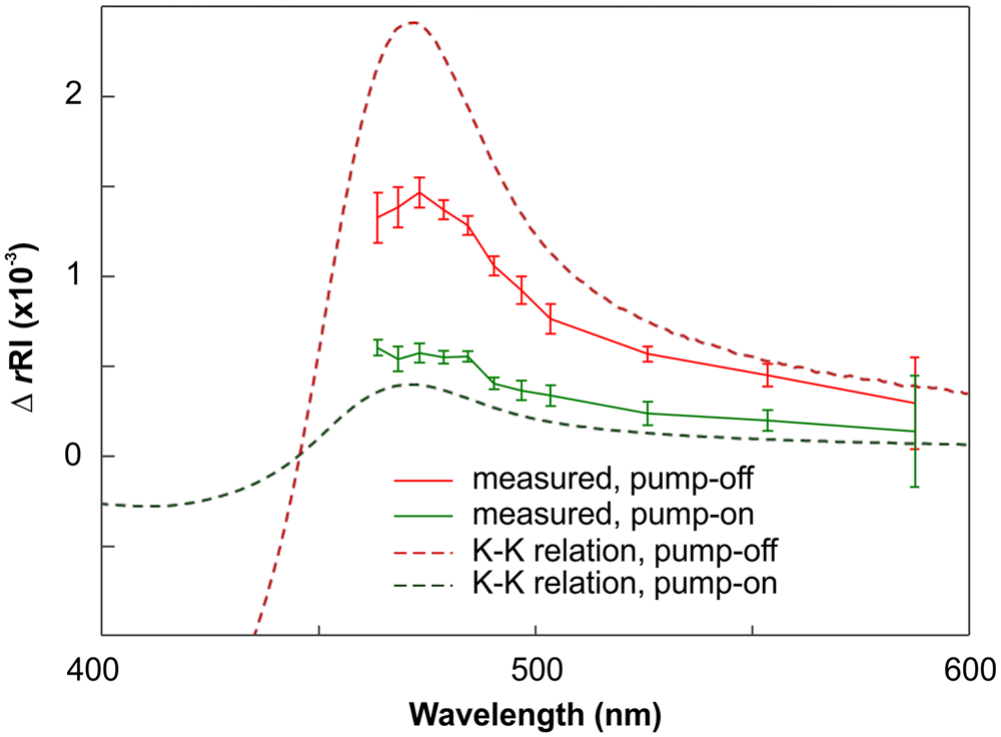
Comparison between calculated Δ*r*RI trends of the PYP based on the K–K relations (dotted lines) and measurements (solid lines). The solid lines are identical to the inset in Fig. 2b. The red and green curves indicate the case in the absence (pump-off) and the presence (pump-on) of the pump beam, respectively. The *y*-axis offset of the dotted lines is set discretionarily because of the ambiguity arising from the integral forms of the K–K relations.

To explain the results of these measurements, we employ the K–K relations. The K–K relations connect the *r*RI and *i*RI based on the causality of the response functions. The K–K relations allow for calculation of the *r*RI from the *i*RI by

5 $$r{\rm{RI}}(\omega )=1+P{\int }_{-\infty }^{+\infty }\frac{d\omega ^{\prime} }{\pi }\frac{i\mathrm{RI}(\omega ^{\prime} )}{\omega ^{\prime} -\omega }$$

or vice versa. Here, *ω* is the angular frequency of light. Although the K–K relations do not provide an exact quantitative solution due to their inherent infinite-integral form, we are still able to obtain qualitative trends for the *r*RI from the well-known values of the *i*RI. The calculated results are shown in Fig. 3. The overall shape of the expected *r*RI obtained from the K–K relations matches well with the experimental results of both the pump-on and pump-off cases and shows the largest deviation at a wavelength of 470 nm, in agreement with our result. In order to retrieve the reliable *r*RI results from the K–K relations, we find that the nearest absorption peaks should be considered at least. In this works, we expect *i*RI measurements over 250 nm – 2,000 nm is required for reliable *r*RI results, regarding the second absorption peak of PYP (280 nm)^65^ and water absorption peaks in infrared regime^66^.

To provide a more intuitive explanation for the *r*RI deviation resulting from the molecular structure change, we introduce the concept of atomic refraction (AR). Historically, AR studies have tabulated the contribution of individual atoms and atomic bonding to the *r*RI of a molecule, and in this way have been able to closely predict the *r*RI of unknown chemicals^67–69^. By considering the AR as a microscopic version of our preconception about the relationship between the *r*RI and density/mass, the deviation in the *r*RI resulting from molecular structure changes can be explained. Further, the AR implies that the *r*RI results may help to reveal atomic bonding changes that occur during the protein conformational change.

The relation between the CRI and electromagnetic susceptibilities [Eq. (1)]^70^ should be emphasized once again. Because the susceptibilities of molecules strongly relate to their electric and magnetic dipole moments, measurements of the CRI can provide clues to protein structure. For example, Tamasaku *et al*. visualized the electron cloud distributions of diamonds with a resolution of 0.54 Å by using extreme-ultraviolet (103 Å–206 Å) light, and the non-linear susceptibility relationship between X-rays and the chosen frequencies^71^. However, in the current study, the CRI measurements of PYP proteins were performed in solution which makes it challenging to directly translate our CRI measurements into structural changes in the PYP. This is because proteins in solution have arbitrary orientations, which result in the smoothing of directional information. When the directional- and/or polarization-dependent CRIs or the electromagnetic susceptibilities of proteins are measured systematically, they have the potential to provide more useful information on the structure of proteins.

## Conclusions

In this work, we presented a method to precisely and quantitatively measure the CRIs of photoactive proteins and their excited states over a wide range of wavelengths. Using a QPI equipped with a wavelength-sweeping source and FTLS, the CRIs of the PYP solution were measured for wavelengths ranging from 461–582 nm.

We found a significant difference in the CRI values of PYP for the absence of a pump beam (pump-off case) and the presence of a pump beam (pump-on case) as a function of wavelength; not only for the *i*RI, but also for the *r*RI. We retrieved the refractive index increment values of PYP for both the *pG* and *pB* states. The maximum difference between ${\alpha }_{pG}$ and ${\alpha }_{pB}$ is approx. 0.3 M^−1^ at 470 nm. We also explained the reason for the unexpected deviation in *r*RI by employing the K–K relation and atomic refraction. We expect the extension of measurable wavelength in the UV regime will help the direct examination of the CRI changes in *pB* states.

The present method measures both the *r*RI and *i*RI values of photoactive proteins simultaneously over a wide wavelength range, and it will be useful for real-time measurements as well as adding to the body of comprehensive, precise, and quantitative information on light-matter interactions. We also expect the present method to see widespread application in measuring the CRIs of photoactive proteins in various fields, including structural biology, chemistry, medical science, and pharmacy. Furthermore, precise measurement of the CRI can provide insight into the application of photoactive proteins in the field of material science and optics.

## Methods

### Sample preparation

The purified PYP solution (see Supplementary Information) was mixed with PMMA microspheres having a diameter of 100 μm (74214 FLUKA, Sigma-Aldrich, Inc.) that had been washed three times with distilled water. Ten microliters of the mixture were sandwiched between the coverslips, and the edges were sealed with epoxy adhesive.

### CRI extraction by Mie theory fitting

To determine the CRI of the PYP solution, we performed nonlinear fitting of the measured FTLS signals using the Mie theory (Fig. 1e). The solution describes the scattering of an electromagnetic plane wave by a homogeneous sphere. We used three fitting parameters: the *r*RI (*n*) and the *i*RI (*κ*) of the PYP solution, and the diameter of the PMMA microspheres. For a robust and automated fitting analysis, the angle-resolved light scattering signals were prepared as a function of $n\,\sin \,\theta$ instead of *θ* in order to ensure the same scale for the horizontal axes while adjusting the *r*RI (n) value because light scattering is dependent on refractive index. During this experiment, we took a photographic image of the microsphere and its surroundings using a CCD camera. We discovered that moving further away from the microsphere’s centre caused the thickness of the PYP solution to increase, causing the CRI to change in response.

Due to the highly oscillatory features of the angle-resolved light scattering curves (Fig. 1e), the direct application of conventional fitting algorithms either shows non-convergent results or is dependent on the initial fitting parameters. Instead, we used a process prior to the fitting processes to find appropriate initial fitting parameters by minimizing the total variance of the extreme positions between the measurement and the Mie theory. These initial parameters yield highly reproducible fitting results. Thereafter, we used a nonlinear fitting algorithm (*nlinfit*, a built-in function of MatLab^TM^) with these predetermined initial fitting parameters. We expect the fitting method can be further simplified by employing global optimization algorithms such as genetic algorithms^72^.

### PMMA microspheres CRI calibration

Mie scattering is highly dependent on the CRI of both the surrounding medium and the homogeneous sphere. In order to measure the CRI of the PYP solution, the CRI of the PMMA microspheres used should be well-known. However, we found that the CRI of polymers such as PMMA can slightly vary product-by-product, depending on the manufacturing procedure. Therefore, the CRI of the PMMA microspheres should be calibrated beforehand.

For the calibration, we used an identical experimental procedure with PMMA microspheres immersed in distilled water, whose CRI value is known^56^. The fitting parameters were the *r*RI, the *i*RI, and the diameter of the PMMA microsphere. The calibrated CRI of the PMMA microsphere was used for the known parameters in the PYP solution CRI measurements.

## Electronic supplementary material

Supplementary Information
