# Supplementary material for: Measurements of complex refractive index change of photoactive yellow protein over a wide wavelength range using hyperspectral quantitative phase imaging
Source: Sci Rep. 2018 Feb 15;8:3064. doi: 10.1038/s41598-018-21403-z (PMC5814402; doi:10.1038/s41598-018-21403-z)
Supplement: Supplementary file 1 — Supplementary Information [file 41598_2018_21403_MOESM1_ESM.pdf]

Supplementary Information (SI)

Measurements of complex refractive index change of photoactive yellow protein over a wide wavelength range using hyperspectral quantitative phase imaging

KyeoReh Lee[1, +], Youngmin Kim[2,3,+], JaeHwang Jung[1], Hyotcherl Ihee[2,3,*], and YongKeun Park[1,*]

[1]Department of Physics, Korea Advanced Institute of Science and Technology, Daejeon 305-701, Republic of Korea.

[2]Center for Nanomaterials and Chemical Reactions, Institute for Basic Science (IBS), Daejeon 305-701, Republic of Korea.

[3]Department of Chemistry, Korea Advanced Institute of Science and Technology, Daejeon 305-701, Republic of Korea.

[*]yk.park@kaist.ac.kr and hyotcherl.ihee@kaist.ac.kr.

[+]These authors contributed equally to this work

Table of contents

Table S1. Specification of pump and probe beams ……………………………………..……(S-4)

Table S2. Refractive index increment ($\partial r\mathrm{RI}/\partial\rho$) of pG and pB states of PYP solution.…….…..(S-4)

Figure S1. Detailed optical setup………………………………………………………….(S-5)

Figure S2. Specification of pump and probe beams ...…………………………………………(S-6)

Figure S3. UV-VIS optical density measurement for PYP….…………………………………(S-6)

Methods

Purifying PYP

The pQE80L-PYP plasmid containing the PYP gene was transformed into *E. coli* BL21 (DE3). Then, a freshly grown colony from the transformed bacterial culture was used to prepare a 50 mL Luria–Bertani (LB) broth seed culture. The seed culture was further used to inoculate 12 L of LB for a large culture at 37°C. When the optical density (O.D.) of the large culture reached 0.6, isopropyl β-D-1-thiogalactopyranoside (IPTG) was added to the culture in order to obtain a final concentration of 1 mM. The temperature was reduced to 18°C, and the culture was incubated overnight. After culturing overnight, the culture was centrifuged at 6000 rpm for 15 min to harvest the cells. The cells were then dissolved in a 20 mM Tris buffer (pH 7.0) containing 150 mM NaCl and 1 mM phenylmethylsulfonyl fluoride (PMSF) and were sonicated for cell disruption. The chromophore precursor (p-coumaric anhydride) was added to the disrupted cell lysate. The mixture was centrifuged at 17,000 rpm for 1 h to separate the cell debris. The supernatant was subjected to nickel affinity chromatography and purified using the gradient elution method. The purified PYP solution was dialyzed with 20 mM Tris-Hcl and a pH 7.0 buffer, and then concentrated. The purity of the concentrated PYP solution was increased with the Hitrap Q column for ion exchange chromatography.

Derivation of the equation for the relaxation time τ of the *pB* state to *pG* state

Because the relaxation times of the intermediate states between *pG* and pB_2 are relatively short, the time-averaged state of excited PYP can be simply regarded as a pure pB_2 state. We simply denote the ground and excited states of PYP as the *pG* and *pB* states, respectively. Then, we can write a kinetic differential equation as follows:

$$\frac{d[pG]}{dt} = -\mathrm{K}_+[pG] + \frac{1}{\tau}[pB],$$
$$\frac{d[pB]}{dt} = +\mathrm{K}_+[pG] - \frac{1}{\tau}[pB]. \quad (1)$$

The total number of PYP should be $[pG] + [pB] = N_0$. K_+ is pumping rate (s^{-1}). τ is the relaxation time (s). $[pG]$ and $[pB]$ are the molar concentrations of the *pG* and *pB* states, respectively. At the equilibrium state, d[pG]/*dt* is same as d[*pB*]/*dt*; therefore, the above equation can be written as $1/\tau = [pG]/[pB]\mathrm{K}_+$. By applying *pB* population ratio, $R = [pB]/N_0$, then $1/\tau$ would be $(1-R/R)\mathrm{K}_+$. The K+ can be written as follows[1]:

$$\mathrm{K}_+ = \frac{\sigma\phi}{h\nu} I = \ln(10)\frac{\varepsilon}{N_A}\frac{\phi}{h\nu} I, \quad (2)$$

where σ is the absorption cross section (m^2), ϕ is the quantum yield, I is the pumping intensity (W/m^2), ε is the extinction coefficient (m^2/mol), N_A is Avogadro's constant (mol^{-1}), and $h\nu$ is the photon energy (J). The absorption cross section (also known as the molar attenuation coefficient) is $\sigma = \ln(10)\,\varepsilon / N_A$.

Thus, we can obtain the following equation:

$$\frac{1}{\tau} = \ln(10)\left(\frac{1-R}{R}\right)\frac{\varepsilon}{N_A}\frac{\phi}{h\nu} I. \quad (3)$$

Since the pump beam has a certain range of wavelength, the equation can be written as follows:

$$\frac{1}{\tau} = \ln(10)\left(\frac{1-R}{R}\right)\frac{\phi}{hcN_A}\int \varepsilon\lambda \frac{\partial I}{\partial \lambda} d\lambda. \quad (4)$$

Table S1. Specification of pump and probe beams

Type	Peak Wavelength (nm)	Peak Intensity (W/mm^3)	FWHM (nm)
**Probe**	461	0.05	4.2
	465	0.10	4.5
	470	0.14	4.8
	476	0.18	5.0
	481	0.22	5.3
	487	0.29	5.5
	492	0.36	5.8
	500	0.44	6.4
	522	0.79	7.7
	549	1.00	10.3
	582	1.05	13.7
**Pump**	445	1824	19

Table S2. Refractive index increment ($\partial r\mathrm{RI}/\partial\rho$) of pG and pB states of PYP solution.

Wavelength (nm)	$\partial r\mathrm{RI}/\partial\rho$ (M^{-1})	
	pG	*pB*
461	3.76 ± 0.04	3.51 ± 0.01
465	3.77 ± 0.03	3.48 ± 0.02
470	3.80 ± 0.02	3.49 ± 0.02
476	3.77 ± 0.02	3.49 ± 0.01
481	3.74 ± 0.01	3.49 ± 0.01
487	3.68 ± 0.02	3.45 ± 0.01
492	3.64 ± 0.02	3.45 ± 0.02
500	3.59 ± 0.02	3.45 ± 0.02
522	3.54 ± 0.01	3.42 ± 0.02
549	3.50 ± 0.02	3.42 ± 0.02
582	3.46 ± 0.07	3.40 ± 0.11

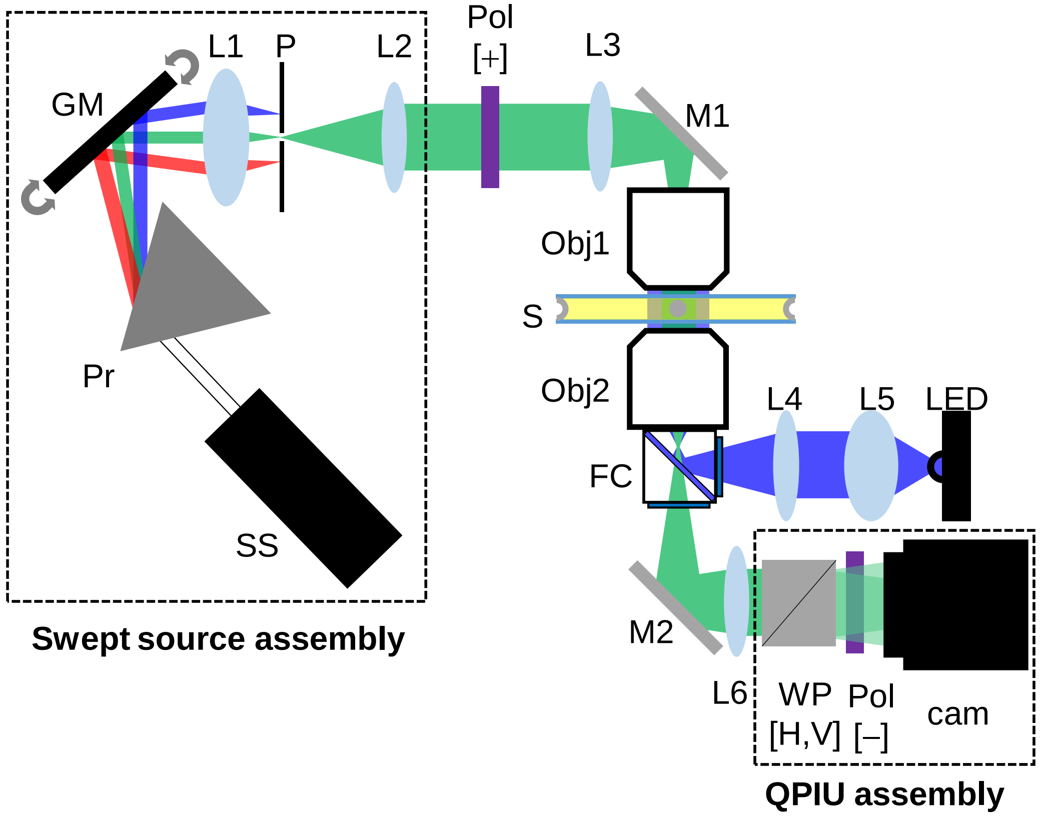

Figure S1. Detailed optical setup. The following are the definitions of the abbreviations: SS, Supercontinuum source (SuperK compact, NKT Photonics); Pr, Prism (PS859, Thorlabs Inc.); GM, Galvano mirror (GVS011/M, Thorlabs Inc.); P, pinhole (P25S, Thorlabs Inc.); Pol, polarizer (LPVISE100-A, Thorlabs Inc.); Obj#, objective lens (Obj1: 40×,0.6 NA, LUCPLFLN 40X, Olympus Inc.; Obj2: 20×,0.4 NA, LMPLFLN 20X, Olympus Inc.); S, sample; FC, filter cube (Excitation filter: FF02-438/24-25, Semrock Inc.; dichroic mirror: FF458-Di01-25x36, Semrock Inc.; emission filter: LP03-458RE-25, Semrock Inc.;); LED, light emitting diode (M455L3, Thorlabs Inc.); WP, Wollaston prism (#68-820, Edmund Optics Inc.); cam, CCD camera (Lt365R, Lumenera Inc.); L#, lens; and M#, mirror. Polarization orientations of polarizers and Wollaston prism are indicated as [H], [V], [+], and [–] which respectively indicate horizontal, vertical, and ±45° linear polarization.

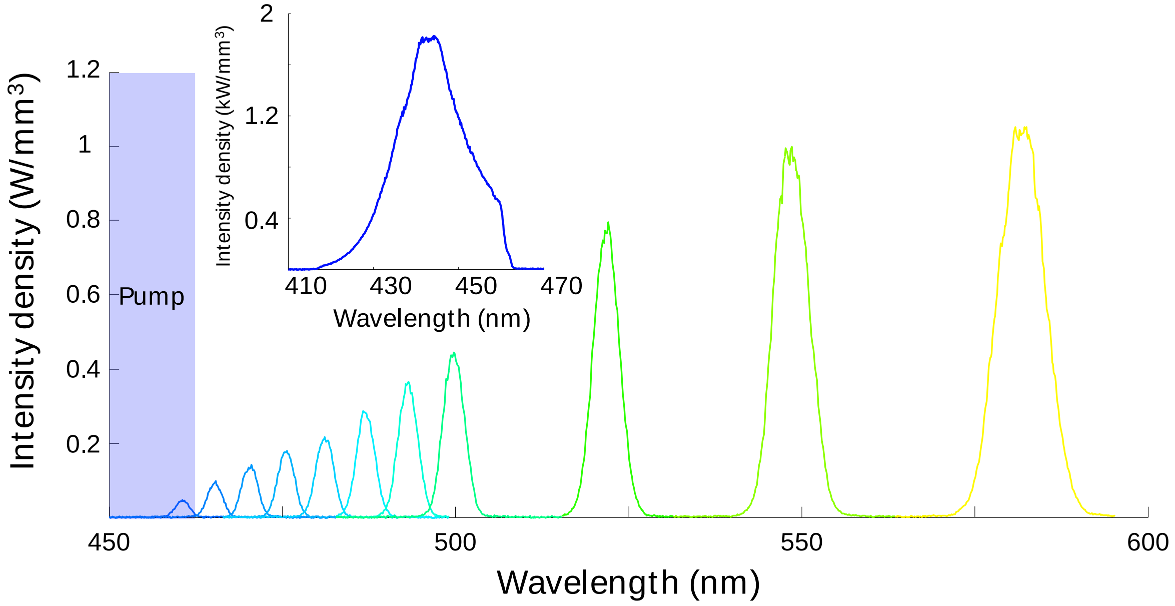

Figure S2. Specification of pump and probe beams. Spectral properties of pump (subset) and probe beam are displayed.

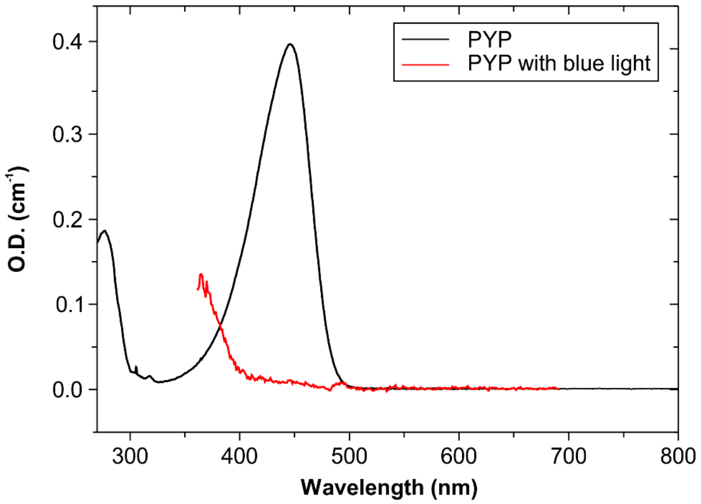

Figure S3. UV-VIS optical density (O.D.) for PYP and PYP with blue light illumination measured by a UV-VIS spectrometer (UV-2550, Shimadzu Inc.).

Reference

1 Strickler, S. J. Citation Classic - Relationship between Absorption Intensity and Fluorescence Lifetime of Molecules. *Cc/Phys Chem Earth*, 18-18 (1981).
